# Development and Validation for Quantification of Cephapirin and Ceftiofur by Ultraperformance Liquid Chromatography with Triple Quadrupole Mass Spectrometry

**DOI:** 10.3390/molecules27227920

**Published:** 2022-11-16

**Authors:** Hari Naga Prasada Reddy Chittireddy, J. V. Shanmukha Kumar, Anuradha Bhimireddy, Mohammed Rafi Shaik, Althaf Hussain Shaik, Abdulrahman Alwarthan, Baji Shaik

**Affiliations:** 1 Department of Engineering Chemistry, College of Engineering, Koneru Lakshmaiah Education Foundation, Vaddeswaram, Guntur 522 502, Andhra Pradesh, India; 2 Aurobindo Pharma Limited, Sanga Reddy, Indrakaran 502329, Telangana, India; 3 Department of Chemistry, College of Science, King Saud University, P.O. Box 2455, Riyadh 11451, Saudi Arabia; 4 Department of Zoology, College of Science, King Saud University, P.O. Box 2454, Riyadh 11451, Saudi Arabia; 5 School of Chemical Engineering, Yeungnam University, Gyeongsan 38541, Republic of Korea

**Keywords:** cephapirin, ceftiofur, cephalosporin antibiotics, beta-lactam ring, reactor rinse

## Abstract

Cross contamination of β-lactams is one of the highest risks for patients using pharmaceutical products. Penicillin and some non-penicillin β-lactams may cause potentially life-threatening allergic reactions. The trace detection of β-lactam antibiotics in cleaning rinse solutions of common reactors and manufacturing aids in pharmaceutical facilities is very crucial. Therefore, the common facilities adopt sophisticated cleaning procedures and develop analytical methods to assess traces of these compounds in rinsed solutions. For this, a highly sensitive and reproducible ultra-performance liquid chromatography with triple quadrupole mass spectrometry (UHPLC-MS/MS) method was developed for the analysis of Cephapirin and Ceftiofur. As per the FDA guidelines described in FDA-2011-D-0104, the contamination of these β-lactam antibiotics must be regulated. The analysis was performed on an XBridge C_18_ column with 100 mm length, 4.6 mm diameter, and 3.5 µm particle size at an oven temperature of about 40 °C. The mobile phase was composed of 0.15% formic acid in water and acetonitrile as mobile phases A and B, and a flow rate was set to 0.6 mL/min. The method was validated for Cephapirin and Ceftiofur. The quantification precision and accuracy were determined to be the lowest limit of detection 0.15 parts per billion (ppb) and the lowest limit of quantification 0.4 ppb. This method was linear in the range of 0.4 to 1.5 ppb with the determination of coefficient (R^2^ > 0.99). This sensitive and fast method was fit-for-purpose for detecting and quantifying trace amounts of β-lactam contamination, monitoring cross contamination in facility surface cleaning, and determining the acceptable level of limits for regulatory purposes.

## 1. Introduction

Cross-contamination among production lines is a critical concern in drug manufacturing, as it can subject both patients and workers at the risk of adverse health effects [1]. Various regulatory bodies in the U.S., Europe, and others are tightening regulations to increase safety and control exposure as the global pharmaceutical markets grow and drugs become increasingly more potent. Tablets or a complex combination of drugs are generally manufactured in large production plants to keep costs low and manufacturing efficient, production lines for a range of different active pharmaceutical ingredients (APIs) are often run in parallel. However, it increases the risk of cross contamination, where active ingredients from one line can be carried across to the other—through the contaminated equipment, air, or via workers’ clothing. If certain sensitizing compounds, such as penicillin and β-lactam antibiotics, make their way into drug production, they can trigger allergic reactions, even at low levels [2]. The risks range from small infections like a non-pruritic, non-urticarial skin rash, or itchy eyes to dangerous immune responses including fatal anaphylactic reactions [3,4,5,6,7]. In fact, penicillin allergy is the most common cause of drug-induced anaphylaxis, and the allergy accounts for up to 1000 deaths per year [8]. In addition to humans, β-lactams antibiotics’ residues are also found most frequently in milk as these compounds are commonly applied as antibiotics in the management of dairy cattle [9]. These residual antibiotics in the milk can also potentially affect human health in the form of allergies and the development of resistance to bacteria [10,11].

β-lactam antibiotics include the following five classes: penicillins, cephalosporins, penems, carbacephems, and monobactams [12,13]. A β-lactam moiety is typically present in penicillin or other non-penicillin drugs including cephalosporins, carbacephems, and monobactams of antibiotics with a long history in the treatment of a broad range of infectious diseases in humans [14,15]. Excessive misuse of β-lactam antibiotics led to β-lactam resistance; additionally, these substances have several side effects like allergy and toxicity [16,17]. Intake of these drugs causes a potential risk in humans who are hypersensitive to them, an important group of patients that are allergic to penicillin, making up around 10% of the adult population [4]. β -lactam antibiotics contain different molecules with diverse molecular structures which consist of a variety of beta-lactam rings; these can be recognized by the immune system leading to hypersensitivity in some patients [18]. For example, cephalosporin induced hypersensitivity reaction and anaphylaxis in patients with IgE-mediated allergy are reported [19]. Apart from this, the toxic effect of β-lactams on the central nervous system is also well-known; for example, the reports of penicillin mediated disorientation, twitching, somnolence, and myoclonus are available in the literature [20]. In addition to their toxic effects, the compounds of cephalosporins including cephapirin, ceftiofur, and many more are potential contaminants present in the production reactors, which are carried forward into the cleaning solution during the cleaning process. The β-lactam of the cleaning solution is present completely unchanged or as an uncyclized form. Moreover, disposal of β-lactam cleaning solution reaches the river, ocean, agricultural lands, landfills and fish farms via municipal sewage [21,22,23]. Many cases were reported with allergic reactions after the consumption of foods and drugs containing antibiotic residues in the literature [24]. Thus, the monitoring of β-lactam compounds in a cleaning solution of drug production manufacturing facilities is essential. The current FDA’s guidance, non-penicillin beta-lactam drugs [25], directed a test method of 1965 that was not sufficiently sensitive [26].

**Figure 1:**
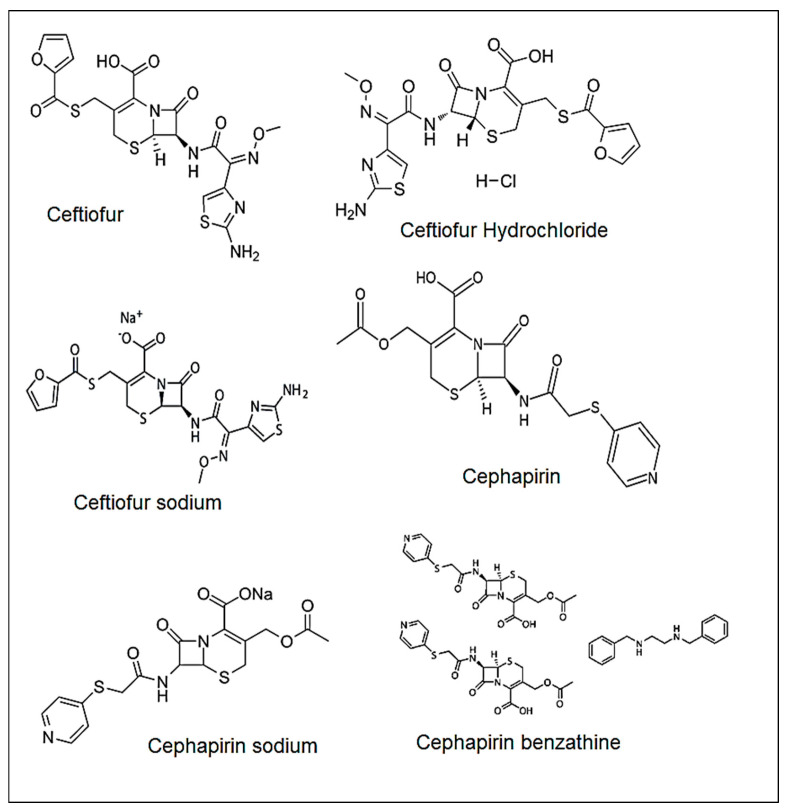
Structures of Ceftiofur, Ceftiofur Hydrochloride, Ceftiofur Sodium, Cephapirin, Cephapirin sodium, and Cephapirin Benzathine.

Many sophisticated and laborious analytical techniques have been utilized for the determination of β-lactam, involving screening methods which include microbial inhibition, radioimmunoassay, enzyme-linked immunosorbent, bioluminescent immunoassay and biosensors [27,28,29,30]. For instance, enzyme immunoassay is conventionally used for the screening of antibiotic residue including aflatoxin M1 or melamine [31]. Apart from this, molecularly imprinted polymer nanoparticles and other metal and metal oxide nanoparticles have been used for the detection and removal of hazardous antibiotic pollutants by enhancing surface area, fast binding kinetics, binding capacity and stability [32,33]. In particular, electrochemical sensing of antibiotics based on various nanomaterials has widely been reported in the current literature [34,35,36]. Unfortunately, using MIP nanoparticles still has a major obstacle, namely inefficient recovery via filtration (significant loss of materials) and centrifugation (time-consuming and laborious). However, they lack specificity and are suitable only when qualitative information is desired. Spectroscopic methods are another alternative method, but the absence of chromophores in β-lactam antibiotics makes them insignificant to UV absorbance; hence, this method needs a suitable derivatization to obtain color [37,38,39]. In addition, many of these methods were developed using conventional high-performance liquid chromatography (HPLC) with UV detection and suffer from a long analysis time [40,41,42]. LC-MS/MS methods were also reported to determine the antibiotics in drug products [43,44] and drug manufacturing surfaces [44]. However, these methods were not developed to determine β-lactam compounds of Cephapirin and Ceftiofur for trace level. Therefore, the development of an easy, rapid, and accurate multi-residue analytical method involving ultra-high performance liquid chromatographic technique is highly required. For example, the eco-friendly QuEChERS technique is broadly applied for the identification of antibiotic residues in various materials including food and other agricultural products [45]. For example, Li et al. have developed a multi-residue analytical technique of QuECHERS in combination with UHPLC-MS/MS to detect several β-lactam antibiotics in aquaculture products [46]. In this perspective, there is a need to develop a sensitive and reproducible method to assess the content of the Cephapirin and Ceftiofur in the solution obtained from the cleaning of reactors used for manufacturing of Ceftiofur free acid, Ceftiofur Hydrochloride, Ceftiofur Sodium, Cephapirin sodium, and Cephapirin Benzathine (Figure 1).

**Scheme 1:**
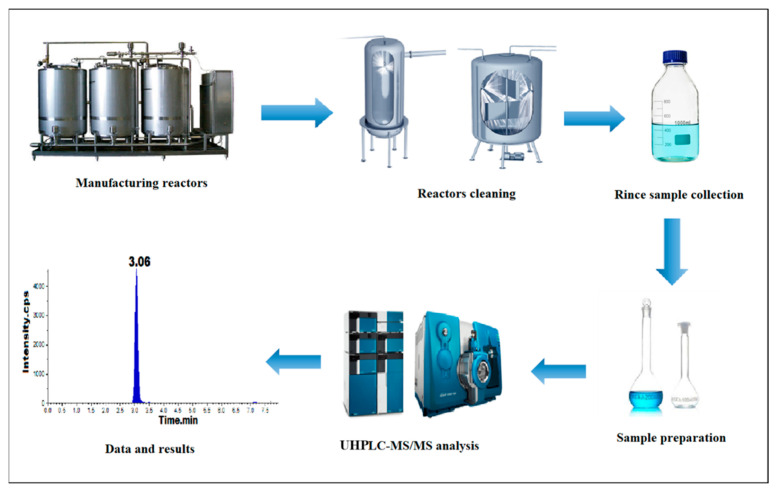
Process flow of cleaning and analysis.

In this present work, we developed a precise, reproducible, and rapid ultrahigh-performance liquid chromatography (UHPLC-MS/MS) method to determine traces of β-lactams in cleaning solutions of the production reactors to prevent the occurrence of cross contaminations in the reactor (Scheme 1). 

## 2. Results and Discussion

### 2.1. Optimization of Mass Spectrometric Parameters

Mass parameters optimization can play a critical role in method development. Interpretation and selection of mass fragments play a key role in the identification and analysis in ppb level impurities analysis. Mass tuning was performed for Cephapirin and Ceftiofur to identify Q1 and Q3 values. Mass tuning was performed by using different ion sources such as atmospheric pressure chemical ionization (APCI) positive, APCI negative and ESI positive, and ESI negative. The m/z 424.0 > 320.0 (for qualification), m/z 424.0 > 292.0 (for quantification) of Cephapirin, and transition ion pairs of m/z 523.8 > 285.0 (for qualification), m/z 523.8 > 241.1 (for quantification) of Ceftiofur MRM mode with ESI ion source and positive ion polarity were finalized; other mass parameters are DP 40, EP 10, CE 22, 25, and CAD medium; GS1 and GS2 are the nebulizer gas 45 and MS temperature 400 °C. The solubility of analytes is checked by using mass-compatible solvents like water, methanol, and acetonitrile. Cephapirin and Ceftiofur are soluble in water, and mass fragments were identified.

### 2.2. Optimization of Chromatographic Conditions

Chromatographic conditions are to be established by using different mass compatible solvents and buffers. Different volatile acidic and basic buffers were used—for example, using ammonia and formic acid with the combination of different solvents like methanol and acetonitrile as mobile phase, different HPLC column chemistries (C_8_, C_18_, phenyl), and different column lengths (250 mm, 150 mm, 100 mm, and 50 mm) and different particle size (5 µm and 3.5 µm). Finally, the method was optimized by using 0.15% formic acid in water and acetonitrile as a mobile phase-A and mobile phase-B, with a gradient program and flow rate of 0.6 mL/min by using an XBridge C_18_ column with 100 mm length, 4.6 mm diameter and 3.5 µm particle size; column temperature is about 40 °C. Cephapirin and Ceftiofur response and ionizations are very good by using the above chromatographic and mass conditions, and retention time was found to be about 3 and 4.4 min.

### 2.3. Method Validation Study

To prove that the method is capable of its intended use, the developed method for the quantification of β-lactam antibiotic traces was validated. The final method was validated in line with ICH guidelines [47]. The validation parameters are system suitability and specificity, limit of detection (LOD), limit of quantification (LOQ), LOQ precision, linearity, method precision, intermediate precision, accuracy, solution stability, and robustness.

### 2.4. Specificity and System Suitability

**Figure 2:**
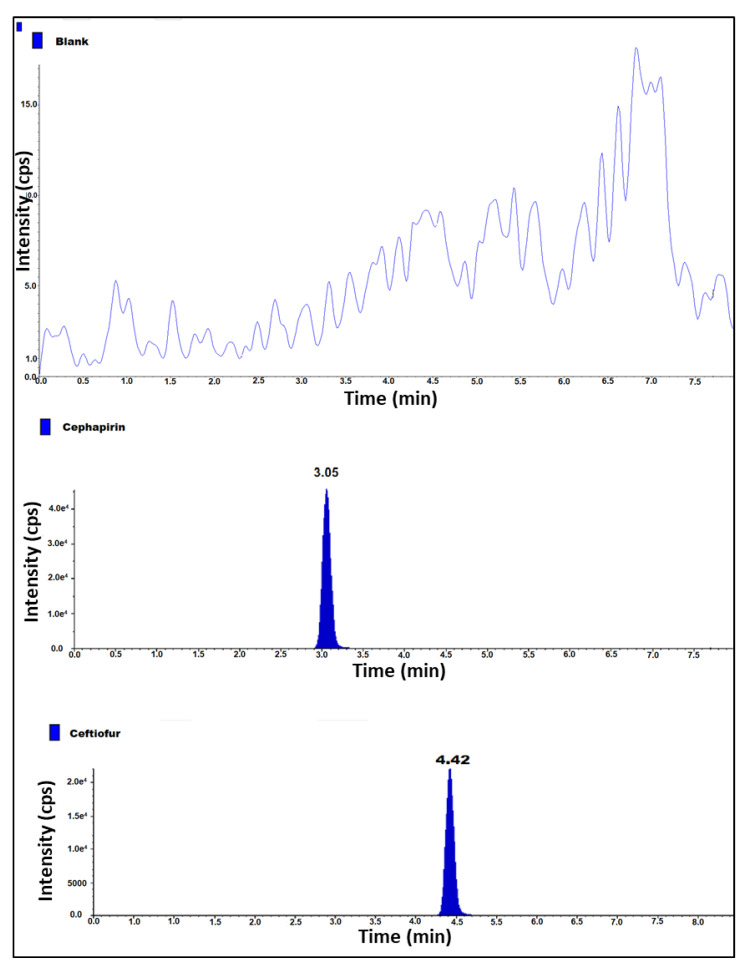
MS/MS chromatogram of blank, cephapirin and ceftiofur standard solution.

As a validation process to ensure the identity of each analyte and analyte retention time, system suitability is performed to check the analyte reproducible response and system efficiency. Specificity followed by system stability was performed, a blank sample was injected, the sample was spiked, an individual standard was prepared in the diluent, and 1 ppb concentration of standards was prepared in each dilution. The peak area percentage relative standard deviation (RSD (%)) of the standard is within the limit, and no interference was observed at the retention time (RT) of Cephapirin and Ceftiofur. The retention times of both the analyte peaks in a spiked sample, standard and individual analytes are eluted at the same retention times. Therefore, this method is specific (Table 1) (Figure 2).

### 2.5. LOD, LOQ and LOQ Precision

**Figure 3:**
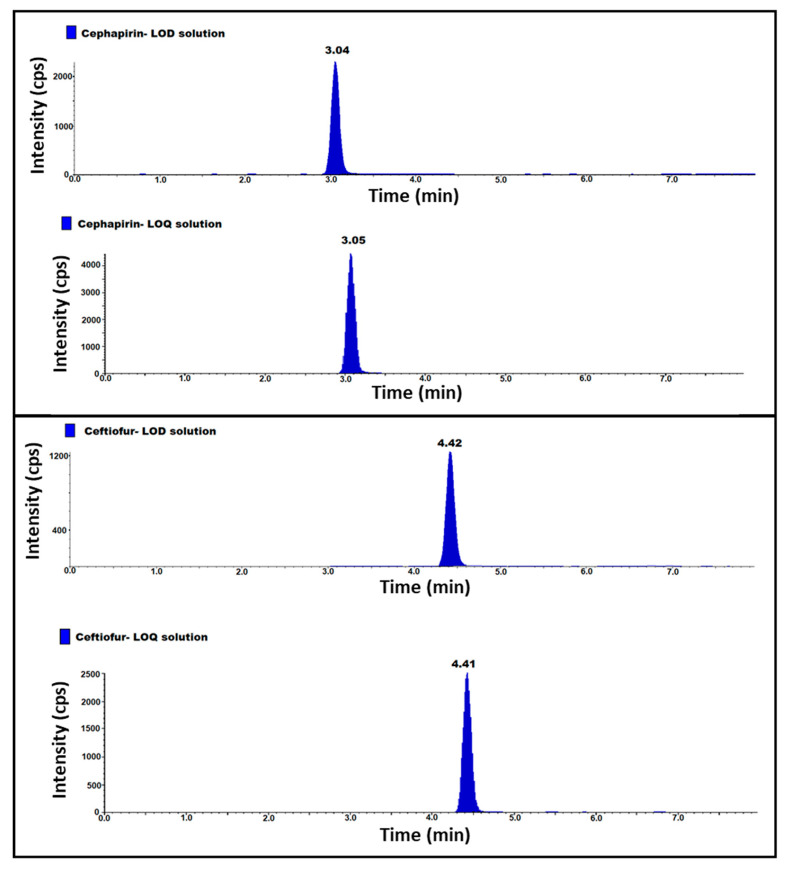
MS/MS LOD and LOQ chromatogram of cephapirin and ceftiofur standard solutions.

Establish a limit of detection (LOD) and limit of quantification (LOQ) by injecting diluted standard solutions, while taking the known concentration of the Cephapirin and Ceftiofur in triplicate, the final concentrations of LOD and LOQ with respect to sample concentration are 0.15 ppb and 0.4 ppb, and the signal-to-noise ratio (S/N) is equal to or greater than 3 for LOD solutions and is equal to or greater than 10 for LOQ solutions. LOQ precision was performed by injecting six replicate injections of LOQ solution. Based on the results, the s/n ratio was greater than 3 for LOD and 10 for LOQ solutions. The area RSD (%) for six replicate injections of LOQ precision is 1.7 and 2.1 for Cephapirin and Ceftiofur (Table 1) (Figure 3).

### 2.6. Linearity and Range

The linearity was established from LOQ to a 150% concentration of cephapirin and ceftiofur (0.4 ppb, 0.5 ppb, 1 ppb, 1.2 ppb, and 1.5 ppb) with respect to sample concentration. Five different known concentrations of LOQ, 50%, 100%, 120%, and 150% are injected in duplicate. The linearity graph peak responses plotted against peak concentrations of cephapirin and ceftiofur evaluated the square of the correlation coefficient (r^2^) and found 0.999 for both of the analytes. Hence, the method was linear (Table 1). 

### 2.7. Method Precision 

The method precision (MP) was established by using a sample. Six samples were prepared as such, and six samples were prepared by spiking each 1.0 ppb of cephapirin and ceftiofur at the specification level and injecting all the solutions. For each preparation, one injection was given to determine the presence of analytes in as such sample’s reproducibility, spiked sample analytes content reproducibility and RSD (%) for the content of cephapirin and ceftiofur. 

As such, the samples do not have any content and the reproducibility of spiked solutions’ content results are repeatable, the obtained content RSD (%) of the spiked solution is 1.0 and 0.6 for cephapirin and ceftiofur peaks. Hence, this method was precise and repeatable (Table 2).

### 2.8. Intermediate Precision

The intermediate precision (IP) was established by repeating MP parameters with different analysts, different days and different lots of columns. The content and RSD (%) of the impurity were determined in sample and spike solutions. As such, the sample solutions do not have the impurity content. The spiked sample solutions (*n* = 6) having RSD (%) were 1.1 and 1.0 for cephapirin and ceftiofur peaks. RSD (%) for preparations (*n* = 12) of MP and IP spiked sample at specification levels less than 20.0. From the results, the method was rugged. (Table 2).

### 2.9. Accuracy

The accuracy was established by spiking cephapirin and ceftiofur into the sample in the range of LOQ to 150% level concentration. The solutions were prepared by spiking cephapirin and ceftiofur into the sample at LOQ, 50%, 100% and 150% (0.4 ppb, 0.5 ppb, 1.0 ppb, and 1.5 ppb concentrations). Each level was prepared in triplicate, and each level was given a single injection. Determine the %recovery of analytes content from spiked sample solutions. The % recovery was observed between 80% to 120% for all the recovery levels. Hence, the method was accurate (Table 2). 

### 2.10. Robustness 

The robustness parameter is used to confirm the ability of the method when slight changes are applied to the final method. By changing the column flow rate plus (+) flow 0.7 mL/min, minus (−) flow 0.5 mL/min and column oven temperature changes to plus (+) column oven temperature at 42 °C and minus (−) column oven temperature at 38 °C, results are compared with the standard and 1.0 ppb spike solution at specification levels of method precision (MP) for retention time (RT) and concentration of cephapirin and ceftiofur. The % difference of cephapirin and ceftiofur content between the results obtained in the method precision and robustness study is less than 5%, and the retention time variation of the analyte ≤ 0.5 min (Table 2).

### 2.11. Solution Stability

Stability studies were performed using a secondary intermediate stock solution of cephapirin, ceftiofur and spiked samples with cephapirin and ceftiofur at 100% concentration levels up to 48 h at ambient laboratory temperature (25 ± 5 °C) and refrigerated condition (2–8 °C). The percent recoveries of primary standard solutions of cephapirin and ceftiofur and spiked samples subjected to stability studies were calculated by comparing them against the freshly prepared primary standard solutions of cephapirin and ceftiofur (Table 2).

Ultra-performance liquid chromatography with triple quadrupole mass spectrometry is a powerful analytical technique for highly specific and quantitative measurements of very low levels of analytes and impurities determination in the pharmaceutical industry. An optimized UHPLC–MS/MS method was developed to determine the cephapirin and ceftiofur content in the cleaning rinse solution of the common reactor in pharmaceutical manufacturing facilities. Since molecular mass is more specific for each compound, no interferences were observed at the retention time of the analyte due to other drug substances or blank. An advantage of this method is the detection of cephapirin and ceftiofur in ppb levels, whereas the reported methods [27,28,29,30,37,38,39,40,41,42,43,44,47] like HPLC, UV Spectrophotometric and LC-MS methods are silent about the content and determination of the cephapirin and ceftiofur. The developed method is simple and direct and lacks any other derivatization process required. The method has the following advantages over the other methods reported. Detection using UHPLC–MS/MS would be a more sensitive and reproducible approach; the proposed method has indicated high accuracy and precision results found during the validation study. The sensitivity was evaluated by the limit of quantification. The LOQ was determined to be 0.4 ppb. This method is as good or superior to that reported in the other papers. Apart from pharmaceuticals, this type of high-performance analytical technique can be used to detect other toxic secondary metabolites generated by the plant and other microbial sources [48,49].

## 3. Material and Methods

### 3.1. Materials and Reagents

Reactor rinsing solutions, Cephapirin, and Ceftiofur have been procured from Jisai Pharma Pvt Ltd. (Hyderabad, India). All chemicals and solvents were of analytical grade, Formic acid and acetonitrile and methanol have been procured from Fischer Chemicals and J.T Baker (Mumbai, India). Water for HPLC grade has been procured from Rankem^®^ (Tiruppur, India) and used for the preparation of all buffers and standard solutions.

### 3.2. Equipment

The traces of β-lactams were determined using a UHPLC system connected with triple quadrupole QTRAP MS/MS equipped with electrospray ionization (ESI) probe make; ABsciex QTRAP 4500 has been used for method development and validation. Analyst software was used to collect and analyze data. For standards and sample weighing, a Mettler Toledo analytical balance was used.

### 3.3. Chromatographic Conditions

Chromatographic conditions were finalized by considering both analytes, based on method development data. The quantification of the compound was achieved with a C18 column (100 × 4.6 mm, 3.5 µm particle size) at an oven temperature of 40 °C. The mobile phase was composed of 0.15% formic acid in water, and acetonitrile as a mobile phase A and B, and a flow rate was set to 0.6 mL/min and deployed the ‘gradient elution program’, which gave the best response within the shortest and acceptable analysis time and column back pressure. The injection volume was 50 µL.

### 3.4. Mass Spectrometer Conditions

**Figure 4:**
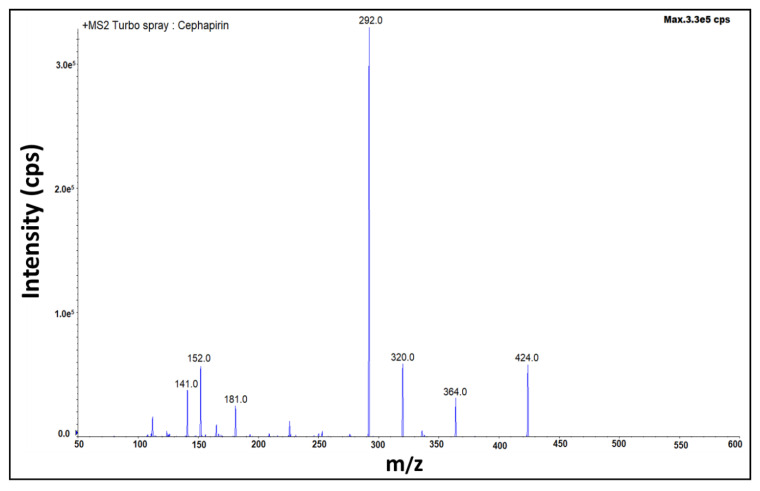
Fragmentation pattern of Cephapirin.

**Figure 5:**
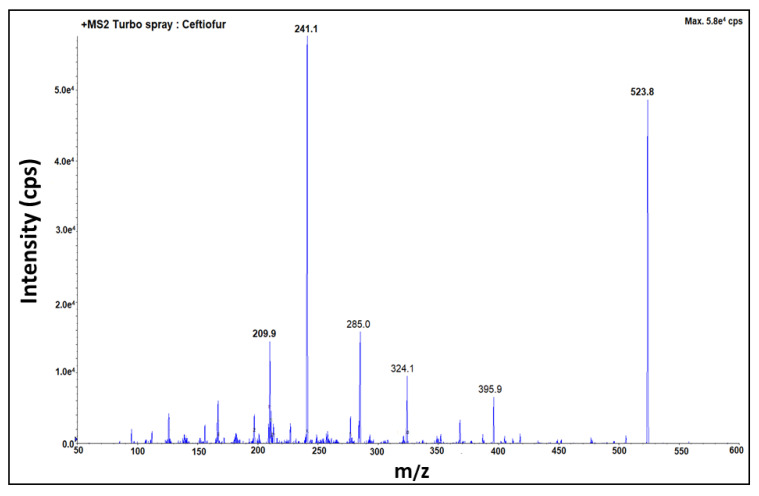
Fragmentation pattern of Ceftiofur.

The MS/MS detector is highly sensitive and reproducible. The MS detector was operated with electrospray ionization (ESI), which is selected as a positive ion source, and multiple reaction monitoring (MRM) selected transition ion pairs of m/z 424.0 > 320.0 (for qualification) and m/z 424.0 > 292.0 (for quantification) of Cephapirin (Figure 4). It also selected transition ion pairs of m/z 523.8 > 285.0 (for qualification) and m/z 523.8 > 241.1 (for quantification) of Ceftiofur (Figure 5). De-clustering potential (DP 40), entrance potential (EP 10) and MS temperature of 400 °C were used as MS/MS detector conditions. Final liquid chromatographic and mass spectrometric method conditions were tabulated. (Table 3).

### 3.5. Preparation of Standard and Test Sample Solutions

Standard and sample concentrations were finalized by using required dilutions, based on the response of impurities during the study by using water as a diluent. Preparation of 1 ppb concentration of Cephapirin and Ceftiofur standards in water used the required dilutions. Directly inject reactor rinse solutions without any dilution. The sample solution was filtered by using a 0.45 µm nylon filter and injected water as a blank. Supplementary chromatograms of the method validation study are provided in Supplementary Files (Figures S1–S14).

## 4. Conclusions

The sensitive, selective, and rapid UHPLC-MS/MS method developed for the identification and quantification of cephapirin and ceftiofur in reactor rinse samples is very sensitive and detects very trace level concentrations. Thus, this new method with advanced technology is capable of the identification and detection of cephapirin and ceftiofur in ppb levels. Considering industrial and guidelines requirements, the method was validated in line with ICH and USP. The method is specific, linear, precise, accurate, rugged and robust. The results of this method demonstrated that reliable data can be obtained in further experiments such as Ceftiofur free acid, Ceftiofur Hydrochloride, Ceftiofur Sodium, Cephapirin sodium, and Cephapirin Benzathine reactor cleaning, and manufacturing aids rinse samples analysis. Therefore, when compared to instrumental analytic techniques and other immunoassays, the method developed in this study can simultaneously detect several structurally different analytes in reactor rinse samples with the advantage of rapidity, low cost and high sensitivity.

## Figures and Tables

**Table 1:** Summary of method validation results.

Validation Parameters	Typical Acceptance Criteria	Results	
		Cephapirin	Ceftiofur
System suitability	RSD (%) for peak area response (*n* = 6) should be ≤15.0.	1.63	1.13
Specificity	Retention time of analyte in all the solutions.	3.0	4.4
	Interference from blank	No interference from blank	
LOD	Concentration of LOD in ppb	0.151	0.152
	S/N value should be ≥3	22	35
LOQ	Concentration of LOQ in ppb	0.412	0.411
	s/n value should be ≥10	55	72
LOQ precision	RSD (%) for six replicate injections of LOQ solution should be ≤15.0%	1.7	2.1
Linearity	Range (ppb)	0.412 to 1.511	0.411 to 1.542
	Square of Correlation coefficient (r^2^) should not be less than 0.99	0.9998	0.9994

**Table 2:** Method validation results summary.

Validation Parameters	Typical Acceptance Criteria	Results	
		Cephapirin	Ceftiofur
Method precision	RSD (%) for six preparations (*n* = 6) of spiked sample at a specification level should be ≤15.0	1.0	0.6
Intermediate precision	RSD (%) for six preparations (*n* = 6) of spiked sample at a specification level should be ≤15.0	1.1	1.0
	RSD (%) for preparations (*n* = 12) of MP and IP spiked sample at a specification level should be ≤20.0	≤20.0	≤20.0
Accuracy	LOQ average recovery (*n* = 3) should be between 70% to 130%.	94.0	96.5
	50% average recovery (*n* = 3) should be between 80% to 120%.	95.2	94.5
	100% average recovery (*n* = 3) should be between 80% to 120%.	96.6	93.5
	150% average recovery (*n* = 3) should be between 80% to 120%.	92.8	90.7
Robustness	Plus (+) flow 0.7 mL/min: spiked sample concentration % difference and retention time	1.1% 2.8 min	1.8% 4.2 min
	Minus (−) flow 0.5 mL/min: spiked sample concentration % difference and retention time	1.4% 3.2 min	1.6% 4.6 min
	Plus (+) oven 42 °C: spiked sample concentration % difference and retention time	2.0% 2.9 min	1.9% 4.3 min
	Minus (−) oven 38 °C: spiked sample concentration % difference and retention time	1.8% 3.1 min	1.7% 4.5 min
Solution Stability	Standard and 100% spiked solution stored at ambient laboratory conditions (25 ± 5 °C) and refrigerated conditions (2–8 °C) were studied for 48 h	Solutions are stable for 48 h	

**Table 3:** Liquid chromatographic and mass spectrometric method conditions.

Parameter	Condition	
**Liquid Chromatography Conditions**		
Mobile phase A	0.15% formic acid in water	
Mobile phase B	acetonitrile	
Auto-sampler temperature	8 °C	
Column temperature	40 °C	
Flow rate	0.6 mL/min	
Injection volume	50 μL	
Gradient program (time in min/mobile phase A)	0/80, 5/5, 5.5/80, 8/80	
Diluent	Water	
Run time	8 min	
**Mass spectrometry conditions**		
Source and Ionization mode	ESI- Positive	
Detection mode	MRM	
MRM transitions (m/z) selected	Cephapirin	Ceftiofur
For qualification	424.0 > 320.0	523.8 > 285.0
For quantification	424.0 > 292.0	523.8 > 241.1
Collision energy (CE)	22	25
De-clustering potential (DP)	40	
Entrance potential (EP)	10	
MS temperature	400 °C	

## Data Availability

Data contained within the article and Supplementary File.

## Supplementary Materials

The following supporting information can be downloaded at: https://www.mdpi.com/article/10.3390/molecules27227920/s1, Figure S1: MS/MS chromatogram of Cephapirin system suitability standard solution; Figure S2: MS/MS chromatogram of Ceftiofur system suitability standard solution; Figure S3: MS/MS chromatogram of Cephapirin LOD solution; Figure S4: MS/MS chromatogram of Cephapirin LOQ solution; Figure S5: MS/MS chromatograms of Cephapirin LOQ Precision; Figure S6: MS/MS chromatogram of Ceftiofur LOD solution; Figure S7: MS/MS chromatogram of Ceftiofur LOQ Precision; Figure S8: MS/MS chromatogram of Ceftiofur Linearity; Figure S9: MS/MS chromatograms of Cephapirin Method precision; Figure S10: MS/MS chromatograms of Ceftiofur Method precision; Figure S11: MS/MS chromatograms of Cephapirin Intermediate precision; Figure S12: MS/MS chromatograms of Ceftiofur Intermediate precision; Figure S13: MS/MS chromatograms of Cephapirin Accuracy; Figure S14: MS/MS chromatograms of Ceftiofur Accuracy.

## Sample Availability

Samples of the compounds are available from the authors on reasonable request.
