# Supplementary material for: Development and Validation for Quantification of Cephapirin and Ceftiofur by Ultraperformance Liquid Chromatography with Triple Quadrupole Mass Spectrometry
Source: Molecules. 2022 Nov 16;27(22):7920. doi: 10.3390/molecules27227920 (PMC9696115; doi:10.3390/molecules27227920)
Supplement: Supplementary file 1 [file molecules-27-07920-s001.zip › molecules-2022093-supplementary.pdf]

Supplementary File

Development and Validation for Quantification of Cephapirin and Ceftiofur by Ultraperformance Liquid Chromatography with Triple Quadrupole Mass Spectrometry

Hari Naga Prasada Reddy Chittireddy [1], J. V. Shanmukha Kumar [1,*], Anuradha Bhimireddy [2], Mohammed Rafi Shaik [3], Althaf Hussain Shaik [4], Abdulrahman Alwarthan [3], Baji Shaik [5]

[1] Department of Engineering Chemistry, College of Engineering, Koneru Lakshmaiah Education Foundation, Vaddeswaram-522 502, Guntur, Andhra Pradesh, India

[2] Aurobindo Pharma Limited, Sanga Reddy, Indrakaran--502329, Telangana, India

[3] Department of Chemistry, College of Science, King Saud University, P.O. Box 2455, Riyadh 11451, Saudi Arabia

[4] Department of Zoology, College of Science, King Saud University, P.O. Box 2454, Riyadh - 11451, Saudi Arabia

[5] School of Chemical Engineering, Yeungnam University, Gyeongsan, 38541, Republic of Korea

* Correspondence: shanmukh_fed@kluniversity.in (J.V.S.K.); mrshaik@ksu.edu.sa (M.R.S.);
Tel.: +91-90-0058-6007 (J.V.S.K.); +966-11-4670439 (M.R.S.)

Validation parameters:

Supplementary File

1. **System suitability and Specificity chromatograms:**

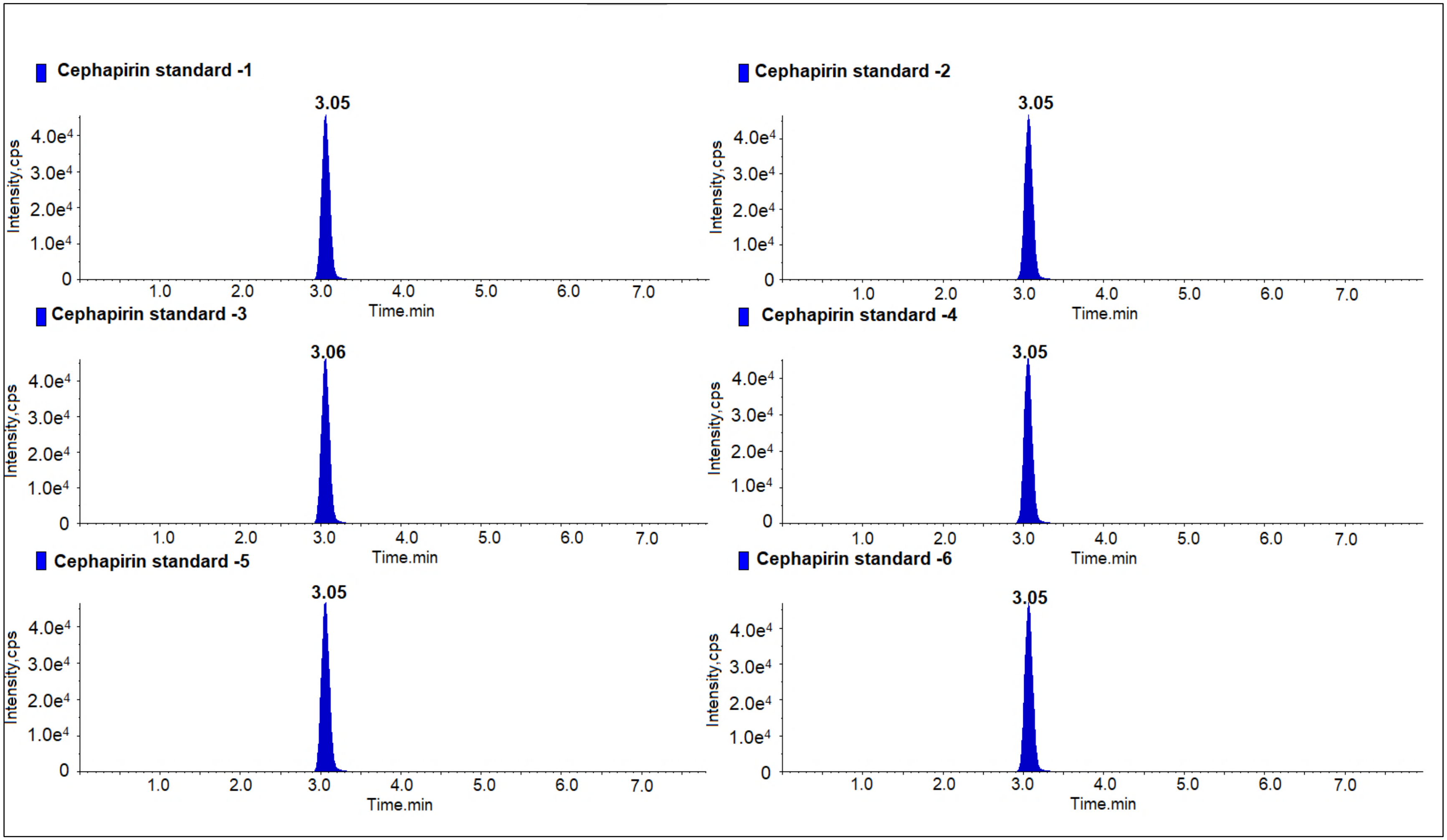

Figure S1: MS/MS chromatogram of Cephapirin system suitability standard solution.

Supplementary File

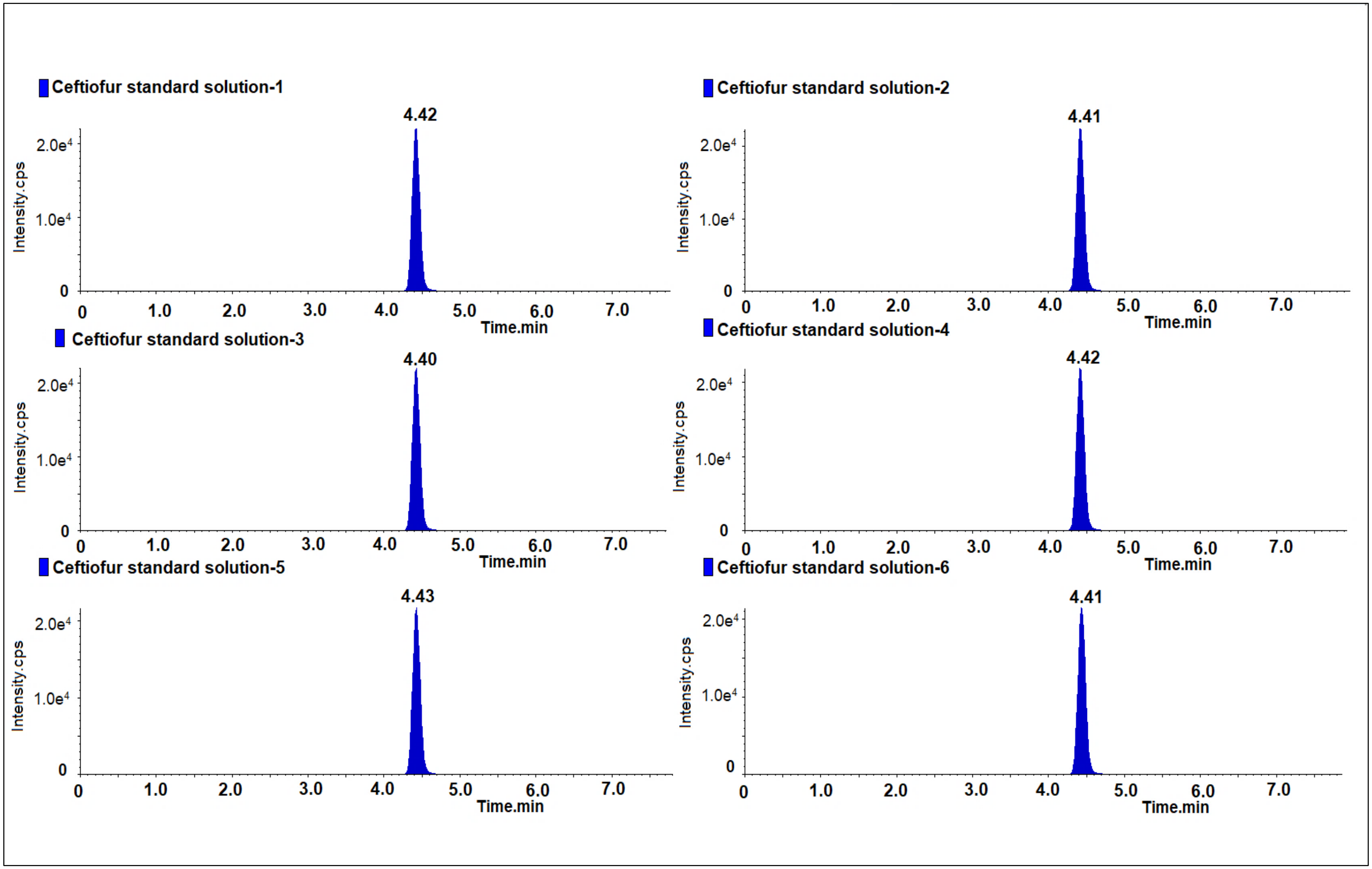

Figure S2: MS/MS chromatogram of Ceftiofur system suitability standard solution.

2. LOD, LOQ and LOQ precision

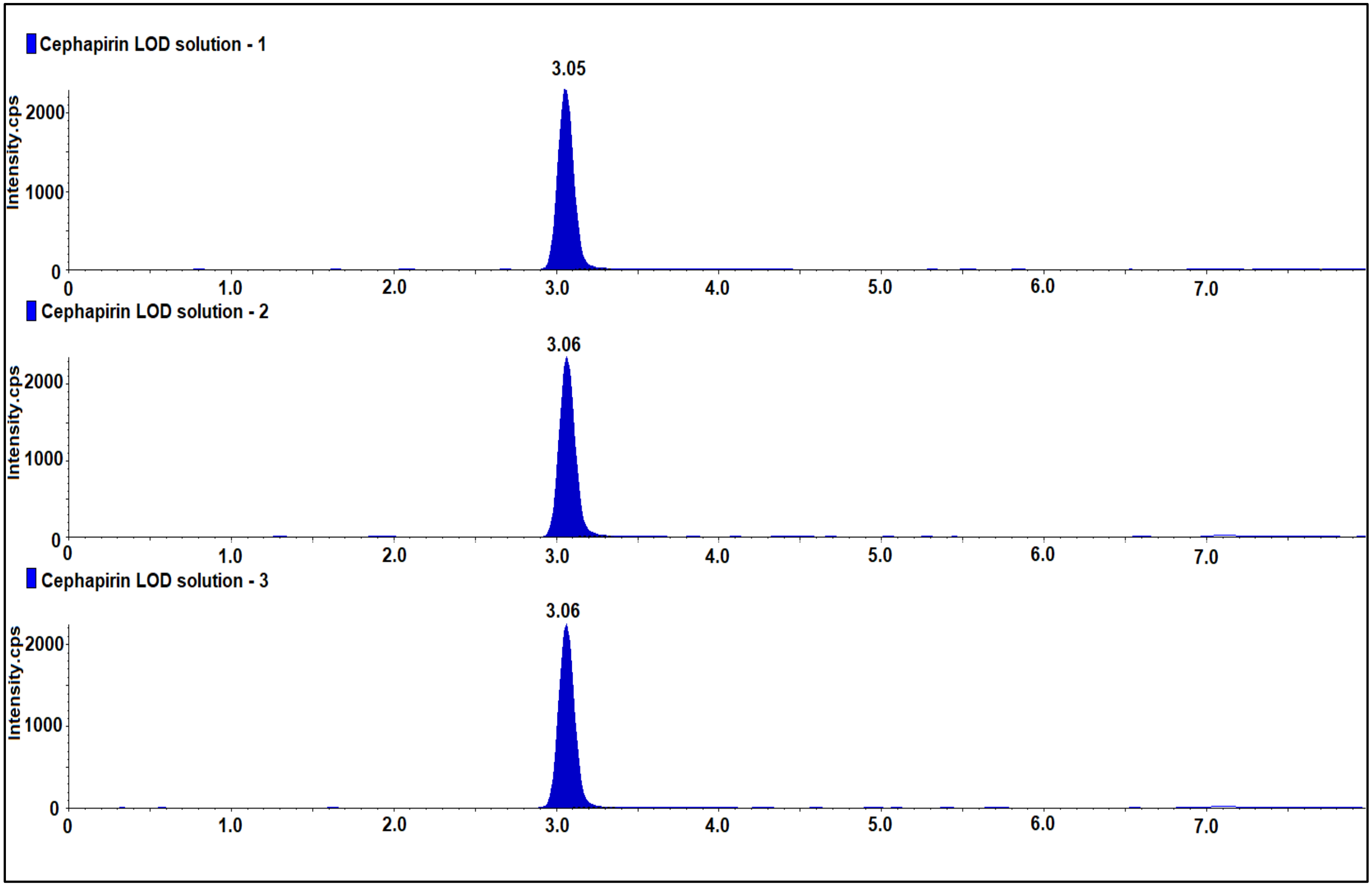

Figure S3: MS/MS chromatogram of Cephapirin LOD solution.

Supplementary File

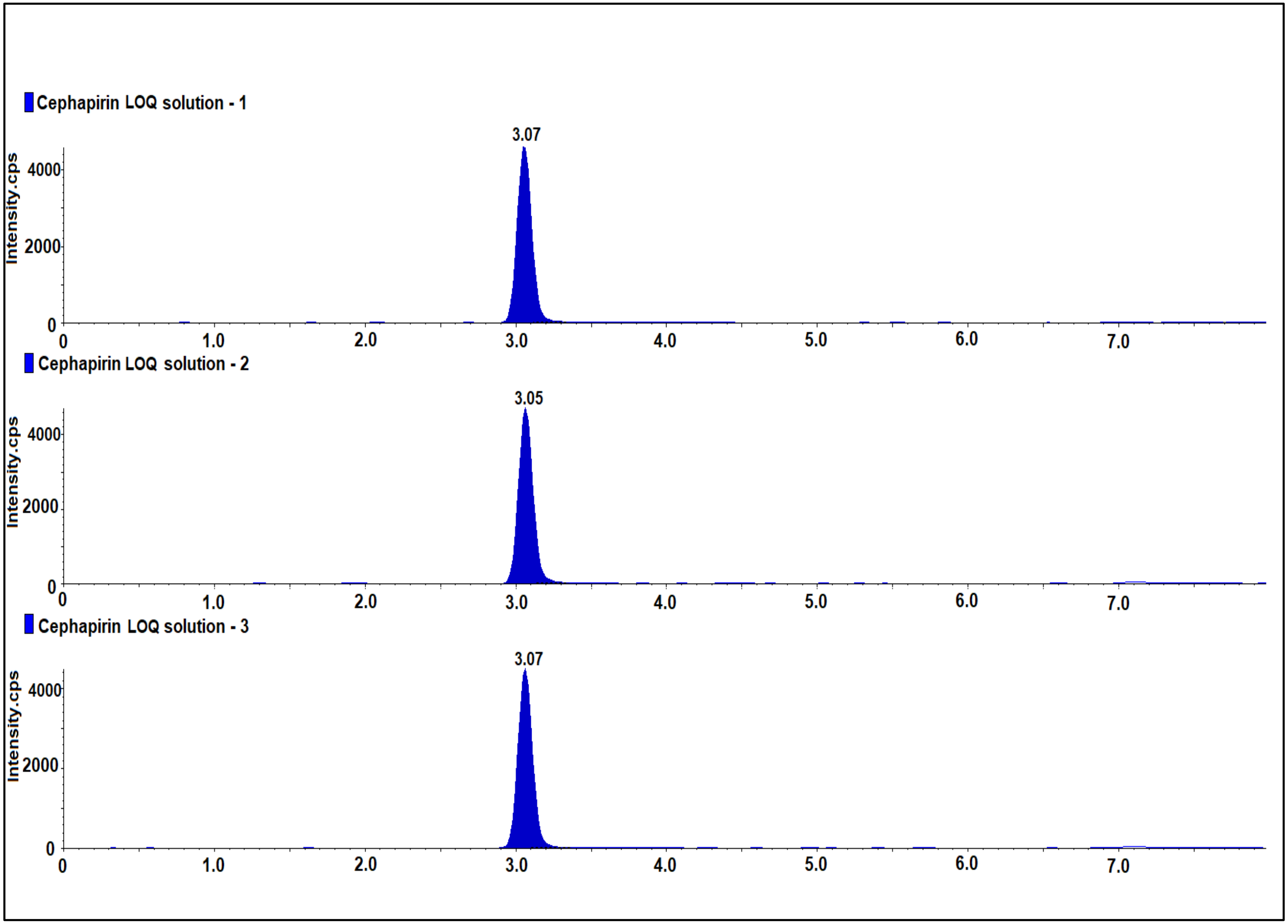

Figure S4: MS/MS chromatogram of Cephapirin LOQ solution.

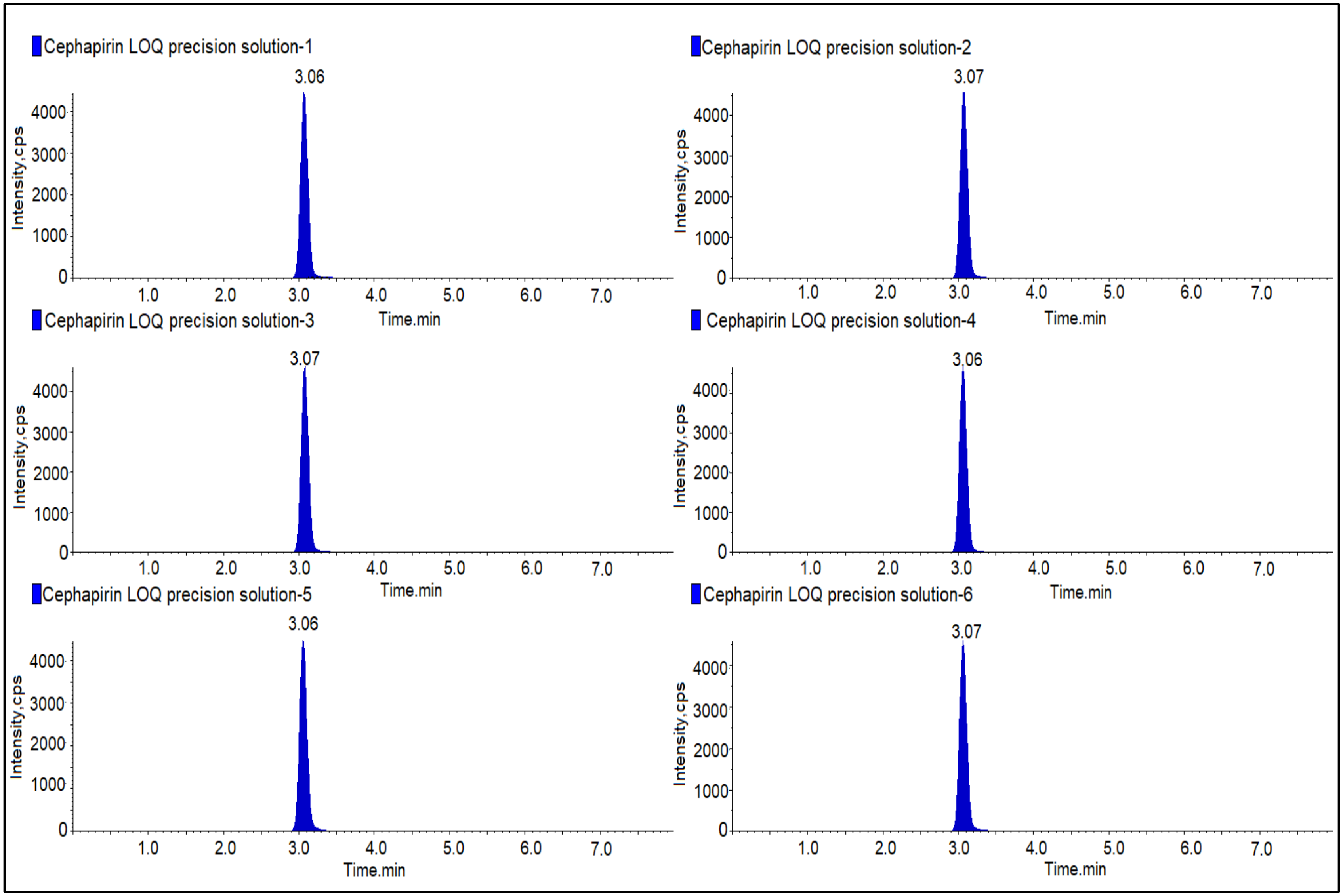

Figure S5: MS/MS chromatograms of Cephapirin LOQ Precision.

Supplementary File

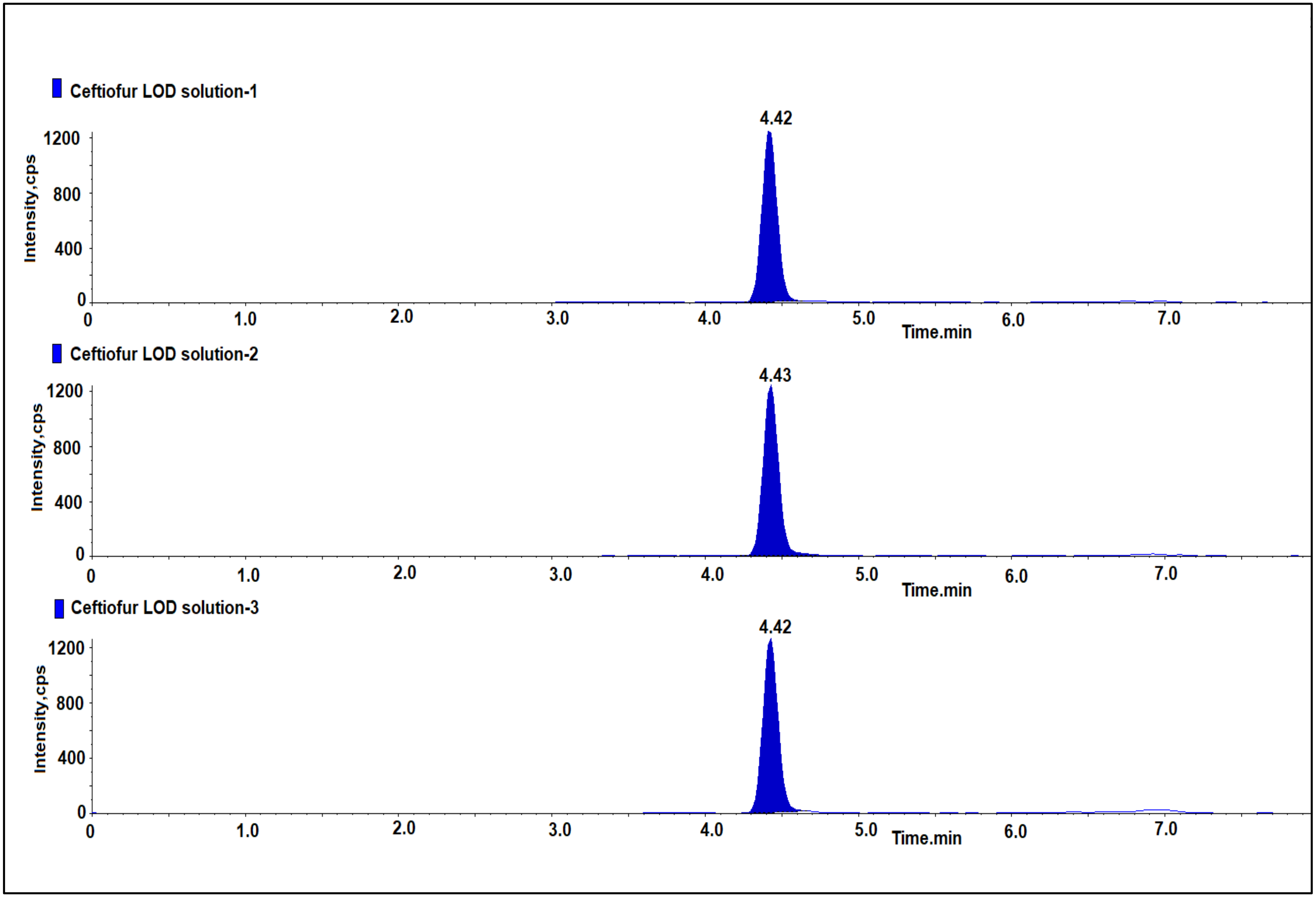

Figure S6: MS/MS chromatogram of Ceftiofur LOD solution.

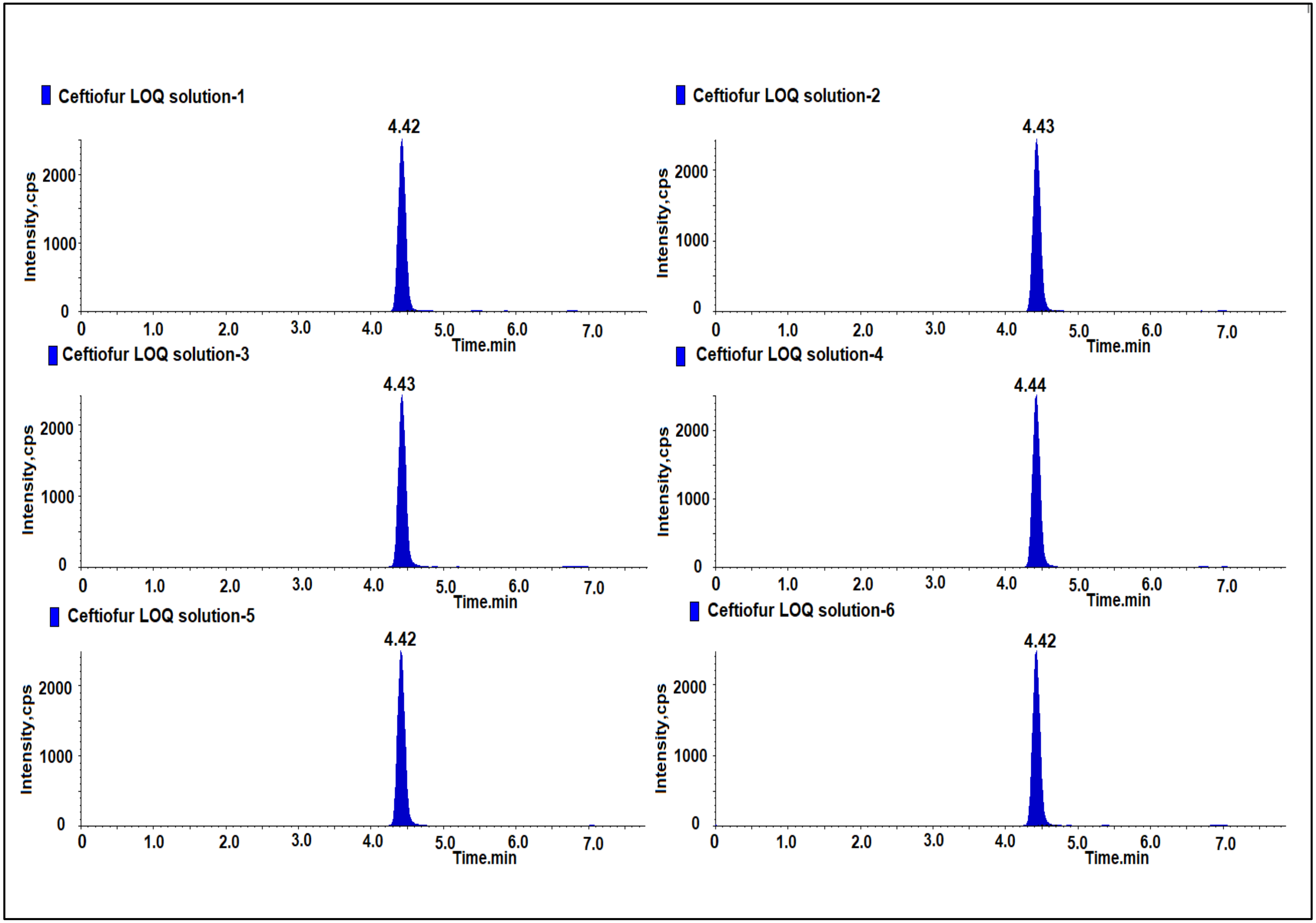

Figure S7: MS/MS chromatogram of Ceftiofur LOQ Precision.

3. Linearity

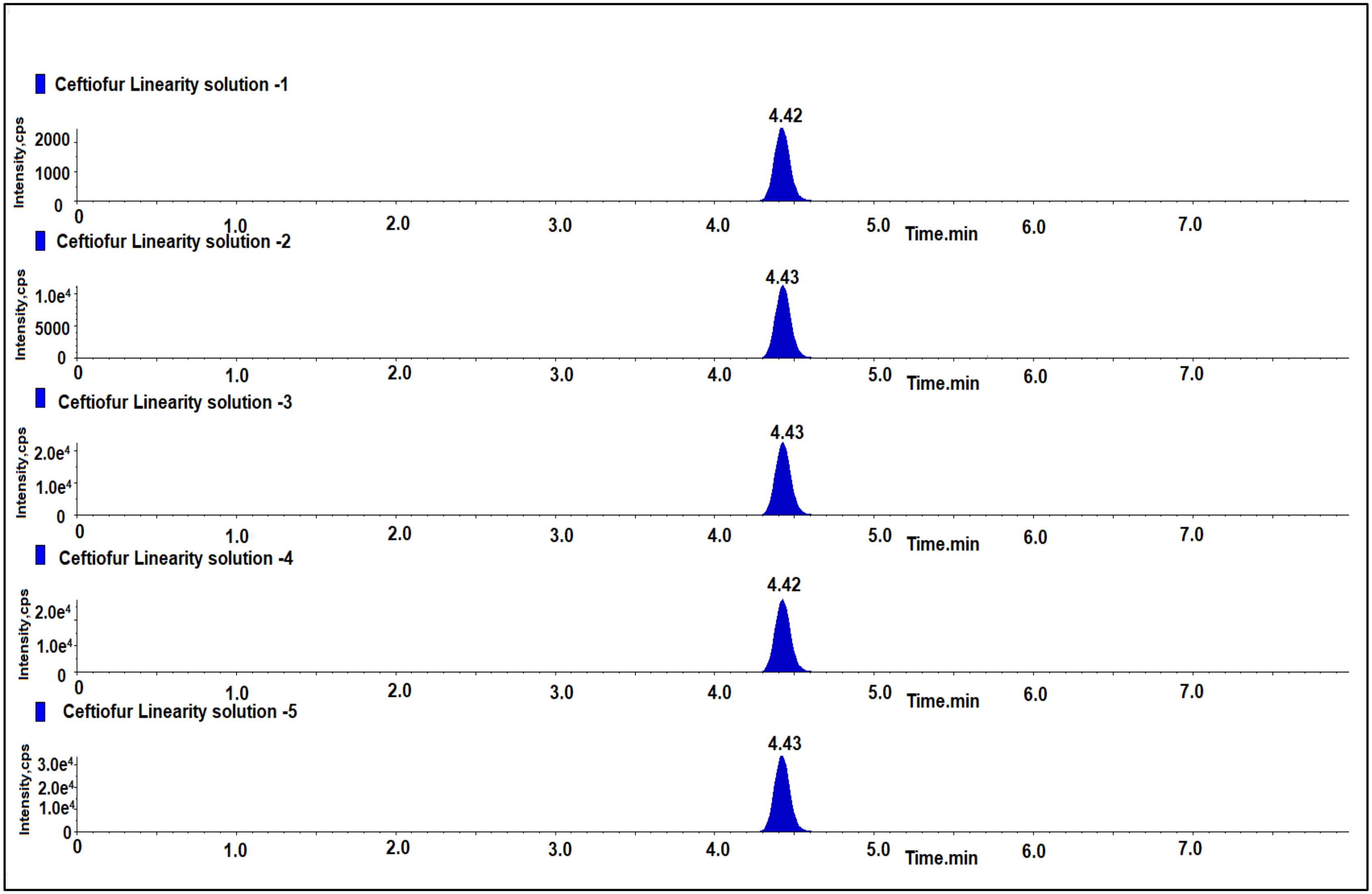

Figure S8: MS/MS chromatogram of Ceftiofur Linearity.

4. Method precision

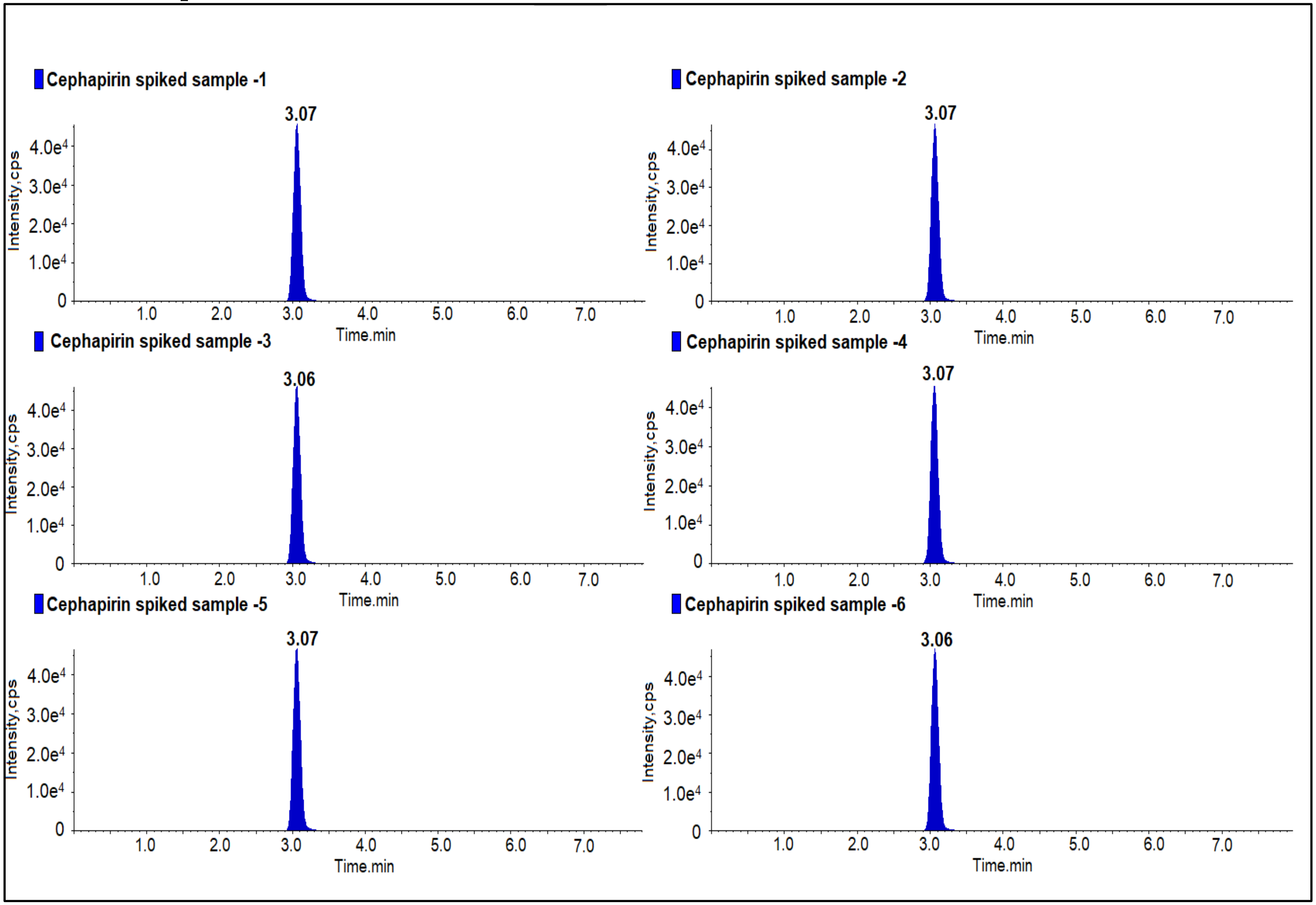

Figure S9: MS/MS chromatograms of Cephapirin Method precision.

Supplementary File

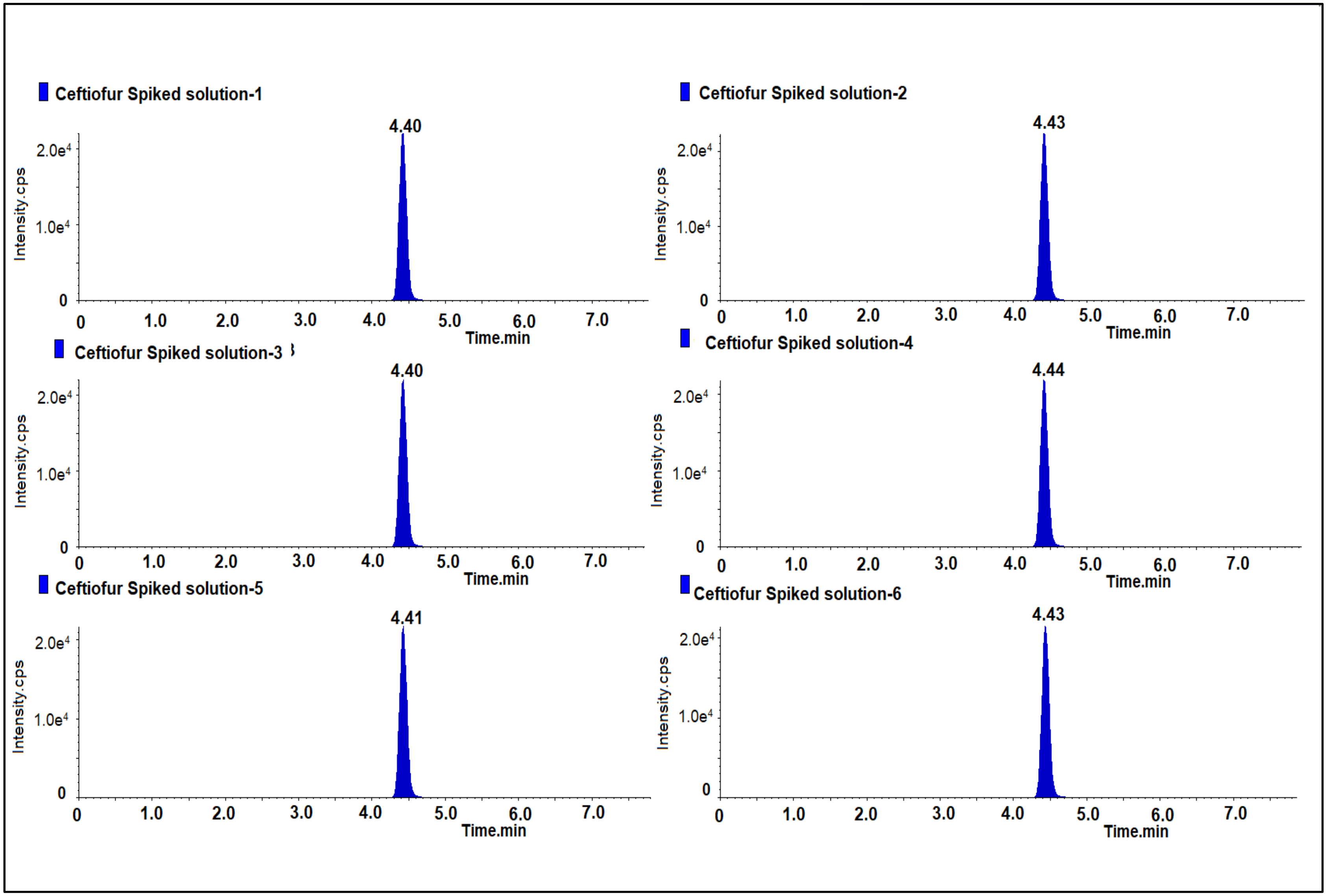

Figure S10: MS/MS chromatograms of Ceftiofur Method precision.

5. Intermediate precision

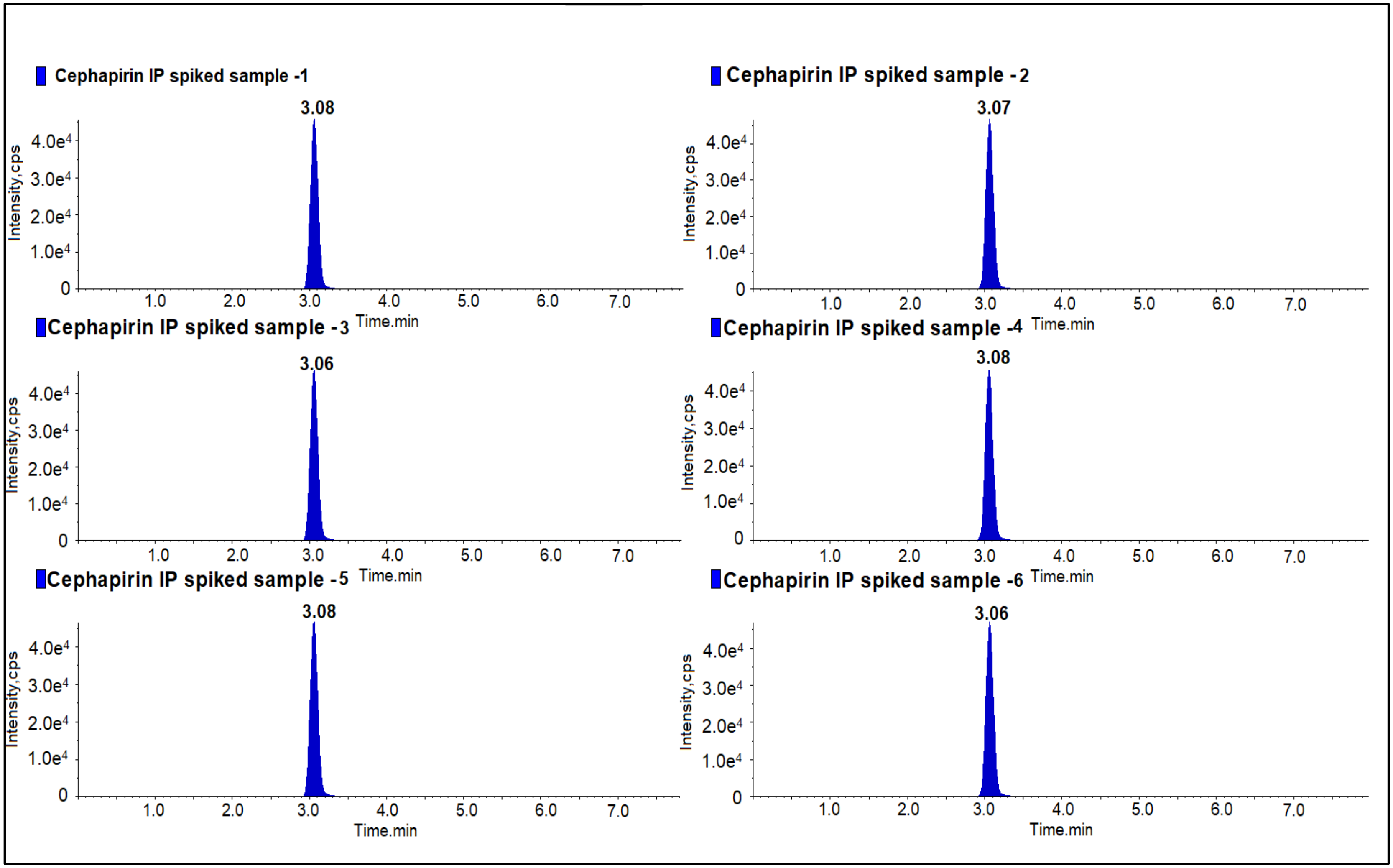

Figure S11: MS/MS chromatograms of Cephapirin Intermediate precision.

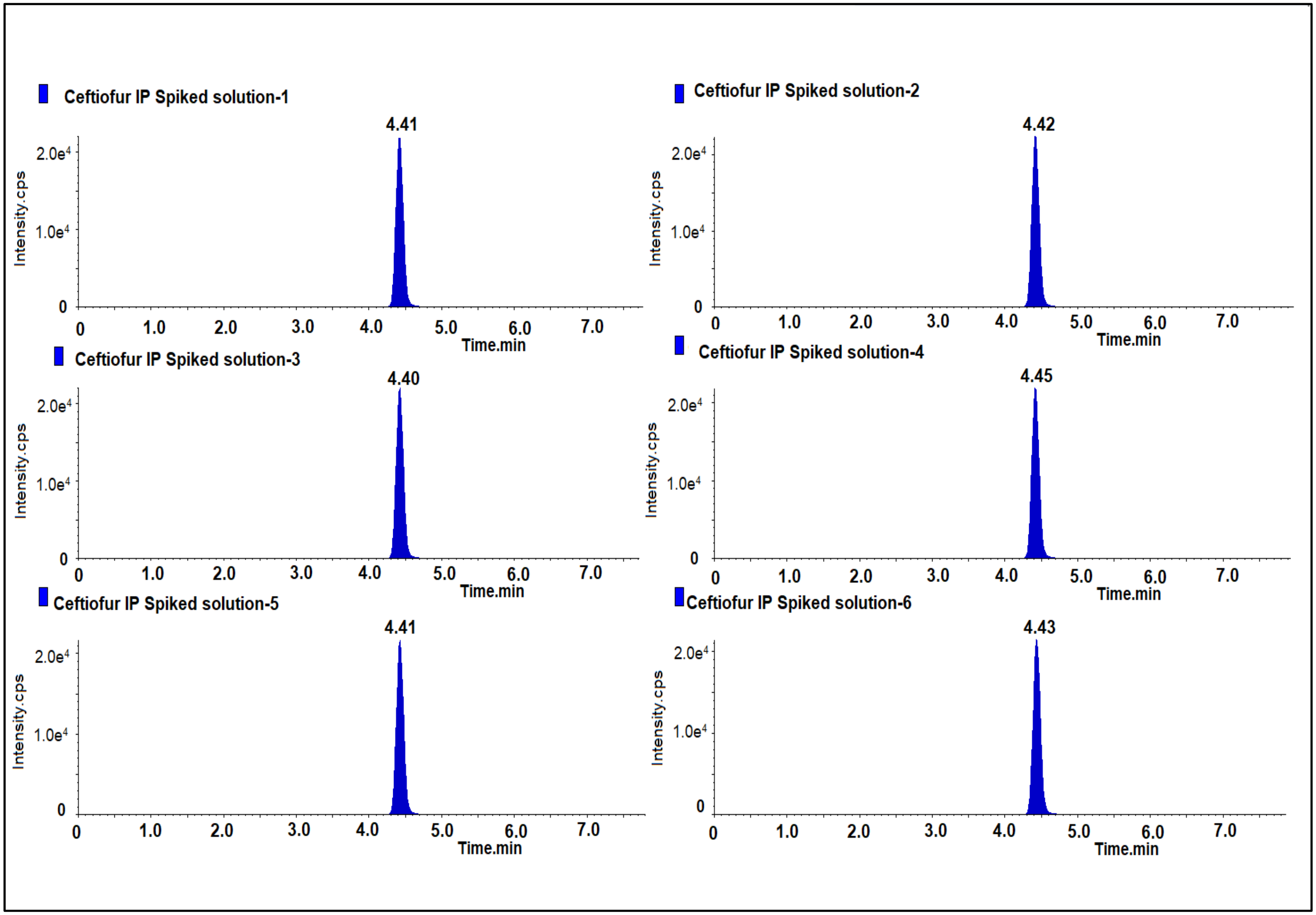

Figure S12: MS/MS chromatograms of Ceftiofur Intermediate precision.

6. Accuracy

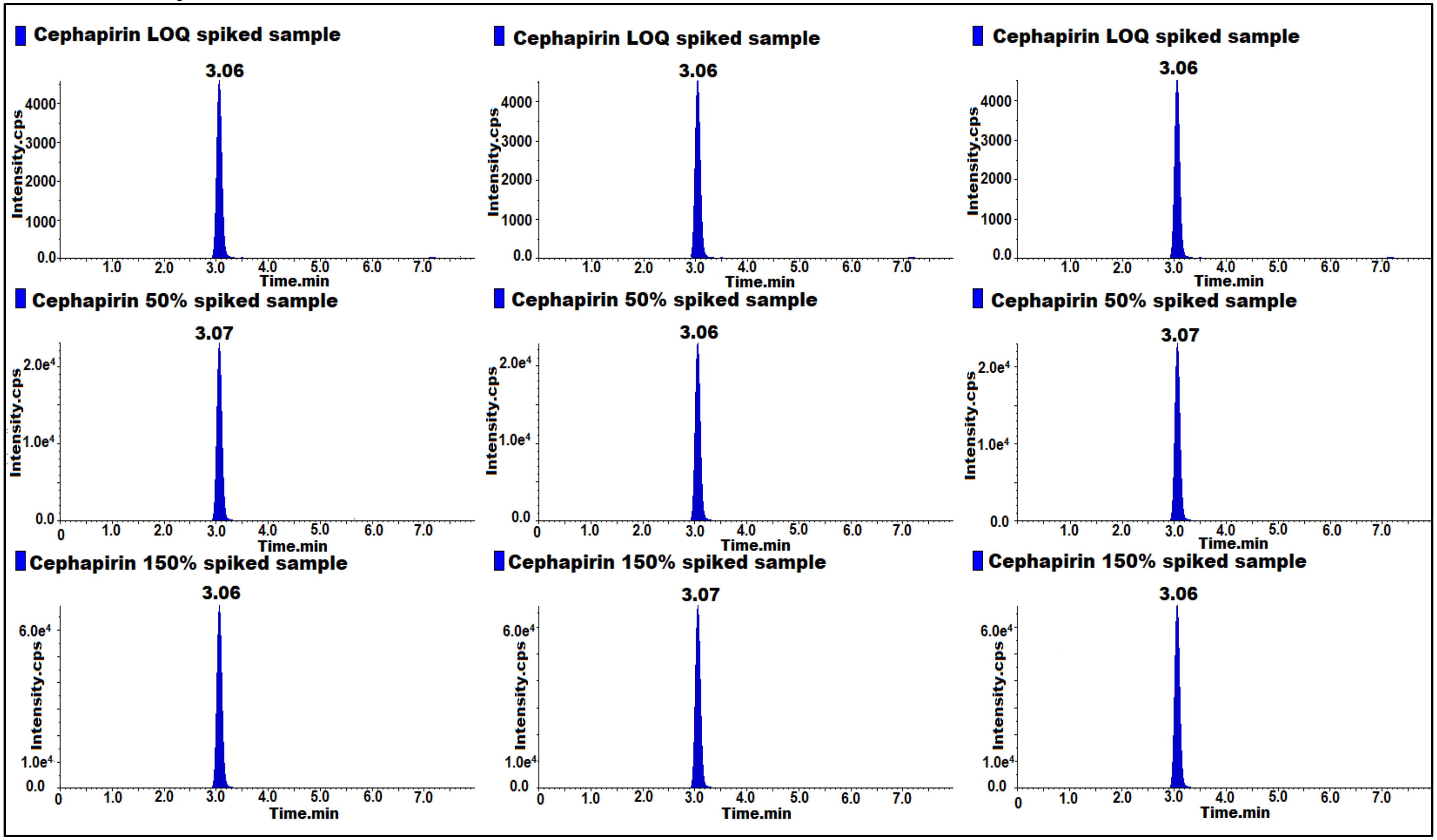

Figure S13: MS/MS chromatograms of Cephapirin Accuracy.

Supplementary File

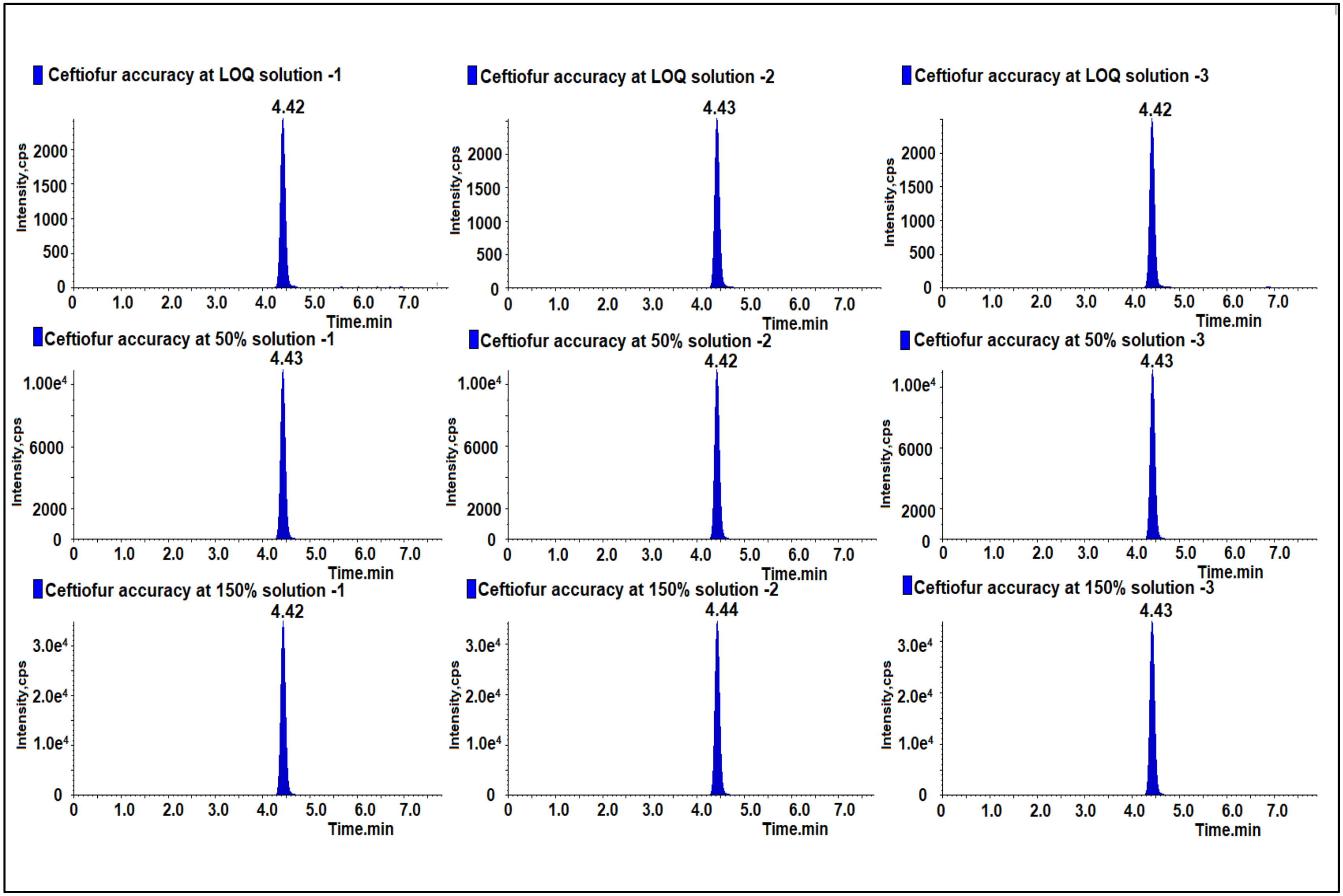

Figure S14: MS/MS chromatograms of Ceftiofur Accuracy.
